# Liuweidihuang Pill Attenuates Early Bleomycin-Induced Pulmonary Fibrosis in Mice and Is Associated with Gut Microbiome

**DOI:** 10.3390/ph19050762

**Published:** 2026-05-13

**Authors:** Yang Zou, Rui-Tao Hu, Qun Yu, Pei-Li Rao, Hong-Yan Cui, Wen-Jing Wei, Xing Cai, Hou-Kai Li, Yun-Hui Shen

**Affiliations:** School of Pharmacy, Shanghai University of Traditional Chinese Medicine, Shanghai 201203, China; hygge_zy@163.com (Y.Z.); huruitao1117@163.com (R.-T.H.); yqhmjk@163.com (Q.Y.); rpl0111@163.com (P.-L.R.); zyydxchy@126.com (H.-Y.C.); 19282130704@163.com (W.-J.W.); cxshutcm@163.com (X.C.); houkai1976@126.com (H.-K.L.)

**Keywords:** Liuweidihuang pill, pulmonary fibrosis, microbiota, inflammatory factors

## Abstract

**Background**: Pulmonary fibrosis (PF) is a chronic, progressive lung disease with limited treatment options. Liuweidihuang pill (LDP), a classical formula for kidney-yin deficiency, has been reported to have anti-inflammatory and anti-oxidative activities, suggesting potential relevance to PF. **Purpose**: This study evaluated whether LDP attenuates bleomycin-induced PF in mice and whether gut microbiota remodeling may contribute to its protective effects. **Methods**: Mice received intratracheal bleomycin followed by LDP gavage. Lung pathology was assessed by hematoxylin–eosin (HE) and Masson staining. Inflammatory cytokines, hydroxyproline (HYP), and α-SMA were measured. LDP and LDP-containing serum were profiled by UPLC-MS. The gut microbiota was analyzed using 16S rDNA sequencing. To further explore whether microbiota-related changes were associated with the protective phenotype, fecal microbiota transplantation (FMT) and probiotic VSL#3 intervention were performed. In addition, LDP-containing serum was tested in a TGF-β1-induced EMT model in A549 cells. **Results**: LDP reduced lung index, inflammatory infiltration, interstitial fibrosis, α-SMA expression, HYP content, and pro-inflammatory cytokine levels in bleomycin-treated mice. These effects were accompanied by gut microbiota remodeling and transcriptomic changes related to inflammation, metabolism, and fibrosis. VSL#3 partially reproduced the protective phenotype, whereas FMT showed limited efficacy. LDP-containing serum had a limited inhibitory effect on EMT inhibited EMT in vitro, suggesting that systemic host responses may contribute to the in vivo effect. **Conclusions**: LDP attenuated early bleomycin-induced PF and was associated with reduced inflammation and gut microbiota remodeling. These findings suggest a possible role for microbiota–host interactions in LDP-associated protection; however, causal directionality, key active effectors, and protein-level pathway validation remain unresolved.

## 1. Introduction

Pulmonary fibrosis (PF) is a major clinical challenge and a common pathological manifestation of interstitial lung diseases (ILDs), and severe cases may result in respiratory failure or death (Kishore et al., 2015 [1]; Wuyts et al., 2020 [2]). A subset of patients with interstitial lung diseases (ILDs) develop progressive pulmonary fibrosis (PPF), defined by worsening symptoms, radiological progression, or physiological decline within one year (Raghu et al., 2022 [3]). Even with standard treatment, the median survival of patients with PPF is only 3.7 years (Montero et al., 2025 [4]). PF arises from diverse causes, including autoimmune disease, drug toxicity, radiation, occupational exposure, allergens (Shenderov et al., 2021 [5]), and infections such as COVID-19 (Fu et al., 2023 [6]). Pro-inflammatory and pro-fibrotic cytokines sustain inflammation and cause irreversible structural damage, including disruption of the alveolar–capillary barrier, thereby driving persistent fibrosis. Many cases remain idiopathic, and the increasing incidence of PF with population aging further highlights the need for effective therapies.

At present, lung transplantation remains the only definitive treatment for advanced PF. Antifibrotic agents, mainly pirfenidone and nintedanib, have improved the treatment of idiopathic PF and other progressive fibrosing interstitial lung diseases by slowing disease progression. However, these drugs cannot reverse established fibrosis or fully halt disease progression, and their long-term use is often limited by adverse drug reactions (Man et al., 2024 [7]; Chianese et al., 2024 [8]). Recent reviews have emphasized that pirfenidone and nintedanib provide clinically meaningful but incomplete benefits, while treatment tolerability remains an important challenge. Therefore, complementary therapeutic strategies are still needed to reduce inflammatory and fibrotic injury and improve long-term disease management.

In traditional Chinese medicine (TCM), the lung is considered the “mother” of the kidney, reflecting the interconnectedness of these vital organs. This concept, known as lung–kidney homology, has guided researchers to explore TCM compounds traditionally indicated for renal disorders in the context of PF management (Xie et al., 2024 [9]). Modern pharmacological research has also suggested that kidney-yin deficiency may be involved in the pathogenesis of PF, potentially through mechanisms related to chronic low-grade inflammation and oxidative stress imbalance (Sullivan et al., 2025 [10]; Yang et al., 2025 [11]; Zuo et al., 2025 [12]). Notably, the biological connection between the lungs and kidneys has a plausible molecular basis. For instance, the renal-derived αKlotho/FGF23 axis, along with inflammatory mediators such as IL-6 and HMGB1, is believed to be involved in the interaction between the lungs and kidneys. These factors can closely link the two organs by regulating inflammatory amplification, oxidative stress, and fibrotic responses (Ravikumar et al., 2016 [13]). Liuweidihuang Pill (LDP), a well-known TCM formula used to treat kidney-yin deficiency, has been investigated for its potential anti-inflammatory and antioxidant properties. Based on modern evidence supporting bidirectional lung–kidney interactions, we hypothesized that LDP may have therapeutic potential in PF. Recent pharmacological studies have shown that LDP can modulate inflammation through multiple components and pathways, including the PI3K-Akt and IL-17 signaling pathways, thereby exerting therapeutic effects in multiple diseases (Perry et al., 2014 [14]; Wang et al., 2024 [15]). Similarly, LDP ameliorates renal interstitial fibrosis by modulating inflammatory cytokines and the TGF-β1/MAPK signaling pathway. Mechanistically, LDP downregulates the NF-κB pathway, which enhances E-cadherin expression, reduces Snail and α-SMA, and increases the endothelial marker CD31 in TGF-β1-induced renal tubular epithelial cells, thereby suppressing epithelial–mesenchymal transition (EMT) and mitigating fibrosis (Pan et al., 2023 [16]; Wang, et al., 2025 [17]; Zhu, et al., 2024 [18]). Furthermore, catalpol, an active compound derived from Rehmanniae radix (the principal herb in LDP), has demonstrated promising anti-fibrotic effects in pulmonary fibrosis (Yang et al., 2020 [19]). Poria also exerts antitumor, antioxidant, anti-inflammatory, and immunomodulatory effects (Li et al., 2023 [20]). Additionally, Shen Qi Wan, a formula similar to LDP, has been shown to attenuate EMT (Lin et al., 2023 [21]). In summary, previous studies have indicated that LDP and its related components have anti-inflammatory, antioxidant and anti-fibrotic potential. However, there is still a lack of direct evidence for their role in pulmonary fibrosis, and it remains unclear whether LDP and its components influence remodeling of the intestinal microbiota.

Increasing evidence indicates that gut microbiota participates in pulmonary inflammation and fibrosis through the gut–lung axis (Costa et al., 2022 [22]), and specific microbial communities and metabolites can distinguish PF patients from healthy individuals (Wu et al., 2022 [23]). The intestinal microbiota also undergoes varying degrees of dysbiosis during PF progression at different time points (Gurczynski et al., 2023 [24]). Meanwhile, previous studies have shown that LDP can modulate intestinal microecology and related metabolic processes in several disease settings (Zhang et al., 2025 [25]; Liu et al., 2022 [26]). We therefore hypothesized that LDP might exert a protective effect in PF by remodeling the intestinal microecology. However, current research mainly focuses on the changes within the PF-related bacterial communities themselves (Chen et al., 2025 [27], Li et al., 2024 [28], Mofidi et al., 2017 [29]), or the regulatory role of LDP on the microbiota in other diseases; whether LDP can exert a protective effect in PF by remodeling the intestinal microecology remains to be directly investigated.

Accordingly, this study was designed to evaluate the protective effect of LDP in early bleomycin-induced PF and to explore the microbiota- and host-response changes associated with this phenotype. The LDP intervention experiment assessed the protective phenotype, 16S rRNA sequencing examined gut microbiota remodeling, FMT tested the transferability of LDP-associated microbiota changes, VSL#3 served as a defined probiotic intervention to assess whether microbiota-related modulation could partially reproduce the phenotype, and RNA-seq explored host response pathways associated with LDP treatment.

## 2. Results

### 2.1. LDP Attenuated the Symptoms of Bleomycin-Induced PF

**Figure 1:**
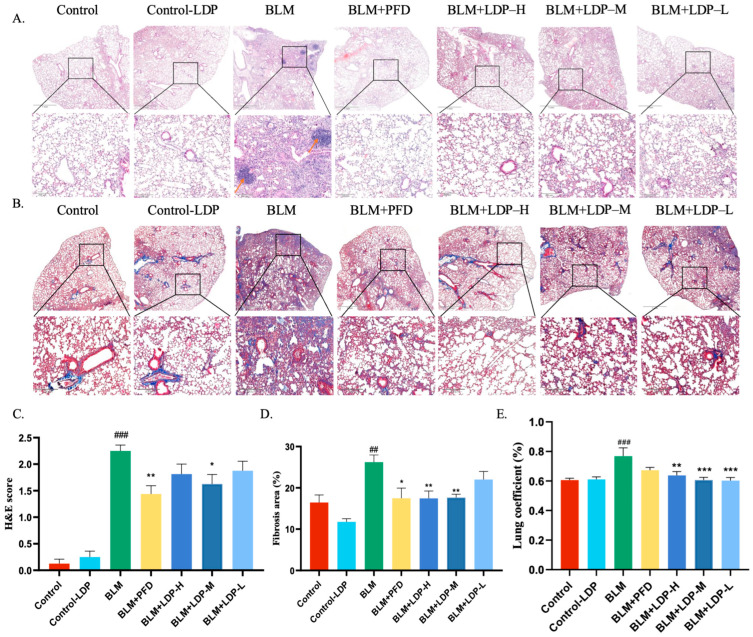
LDP alleviated lung injury and pulmonary fibrosis in bleomycin-treated mice. (**A**,**B**) Representative HE- and Masson-stained lung sections from the CON, BLM, BLM+PFD, BLM+LDP-H, BLM+LDP-M, and BLM+LDP-L groups (*n* = 8). Arrows in (**A**) indicate inflammatory infiltration. Scale bar: 1 mm, 200 μm. (**C**) Histological injury score based on HE staining. (**D**) Fibrosis area quantified from Masson staining. (**E**) Lung coefficient (*n* = 10). * *p* < 0.05, ** *p* < 0.01, *** *p* < 0.001, ## *p* < 0.01, ### *p* < 0.001.

In the LDP intervention study, BLM-induced lung injury produced marked structural abnormalities (Figure S1A). The BLM group showed obvious alveolar damage, including septal edema, alveolar wall thickening, and inflammatory cell infiltration (Figure 1A,C), together with aggravated interstitial fibrosis (Figure 1B,D) and an increased lung mass index (Figure 1E). These changes were significantly alleviated by LDP treatment, with the most prominent effect observed at the medium dose (600 mg/kg), indicating that LDP attenuated bleomycin-induced PF at the early fibrotic stage in this model. Both LDP and pirfenidone improved histopathological injury in bleomycin-treated mice, with comparable efficacy at the optimal dose.

### 2.2. LDP Reduces Inflammatory Factors in the Lung and Serum

**Figure 2:**
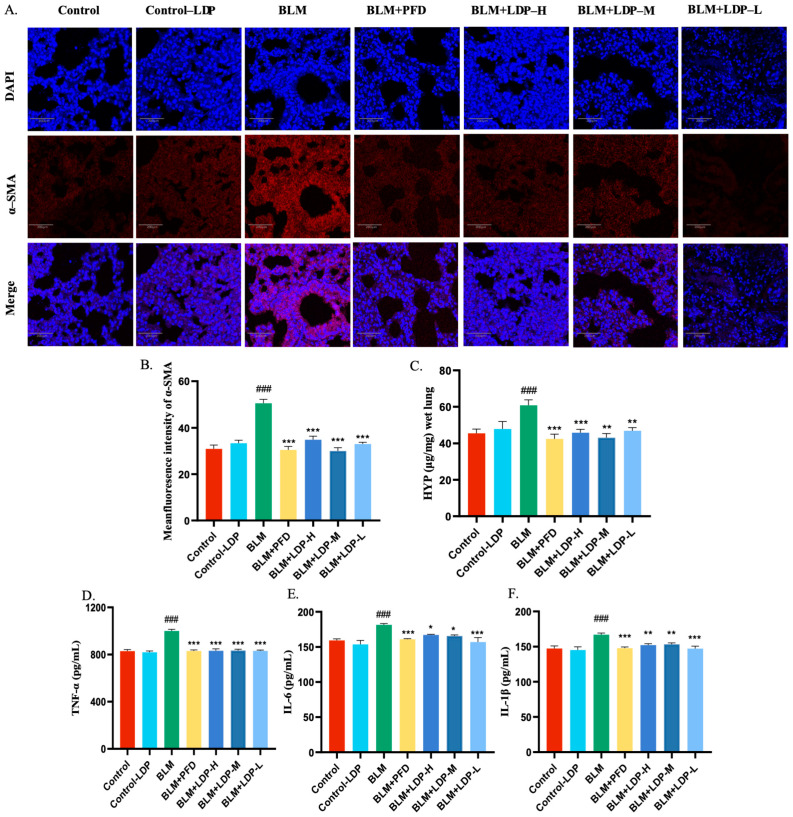
LDP reduced fibrosis- and inflammation-related markers in bleomycin-treated mice. (**A**) Representative immunofluorescence images of α-SMA expression in lung sections from the CON, BLM, BLM+PFD, BLM+LDP-H, BLM+LDP-M and BLM+LDP-L groups (*n* = 8). Scale bar: 200 μm. (**B**) Quantification of α-SMA fluorescence intensity. (**C**–**F**) Serum levels of HYP, TNF-α, IL-6, and IL-1 β (*n* = 10). * *p* < 0.05, ** *p* < 0.01, *** *p* < 0.001, ### *p* < 0.001.

To evaluate the molecular effects of LDP, we measured PF-related markers in serum and lung tissue, including α-SMA, HYP, TNF-α, IL-6, and IL-1β (Figure 2B–F). Because α-SMA is widely used as a fibrosis marker and increases during fibrotic activation, we first examined its expression by immunofluorescence. α-SMA expression in lung tissue decreased after LDP treatment (Figure 2A,B), and HYP followed a similar trend (Figure 2C). BLM induction significantly elevated serum levels of TNF-α, IL-6, and IL-1β, which were reduced following LDP administration (*p* < 0.05; Figure 2D–F).

Among the tested doses, the low-dose LDP group showed the strongest reduction in fibrotic and inflammatory markers, followed by the medium-dose group, whereas the high-dose group showed a weaker response. Notably, the low- and medium-dose LDP groups showed stronger anti-fibrotic and anti-inflammatory effects than the high-dose group, suggesting a bell-shaped rather than a simple linear dose–response relationship. As the medium dose approximately corresponded to the clinically converted dose, its favorable effect may suggest a suitable therapeutic window. Based on previous LDP-related studies, excessively high doses may lead to less favorable regulation of microbiota-related or host protective responses, although this was not directly tested here (Tao et al., 2022 [30]; Lv et al., 2017 [31]). Overall, these findings indicate that LDP reduced inflammation- and fibrosis-related markers in BLM-induced PF mice and attenuated early fibrotic injury.

### 2.3. Qualitative Profiling of LDP and Serum Constituents

**Figure 3:**
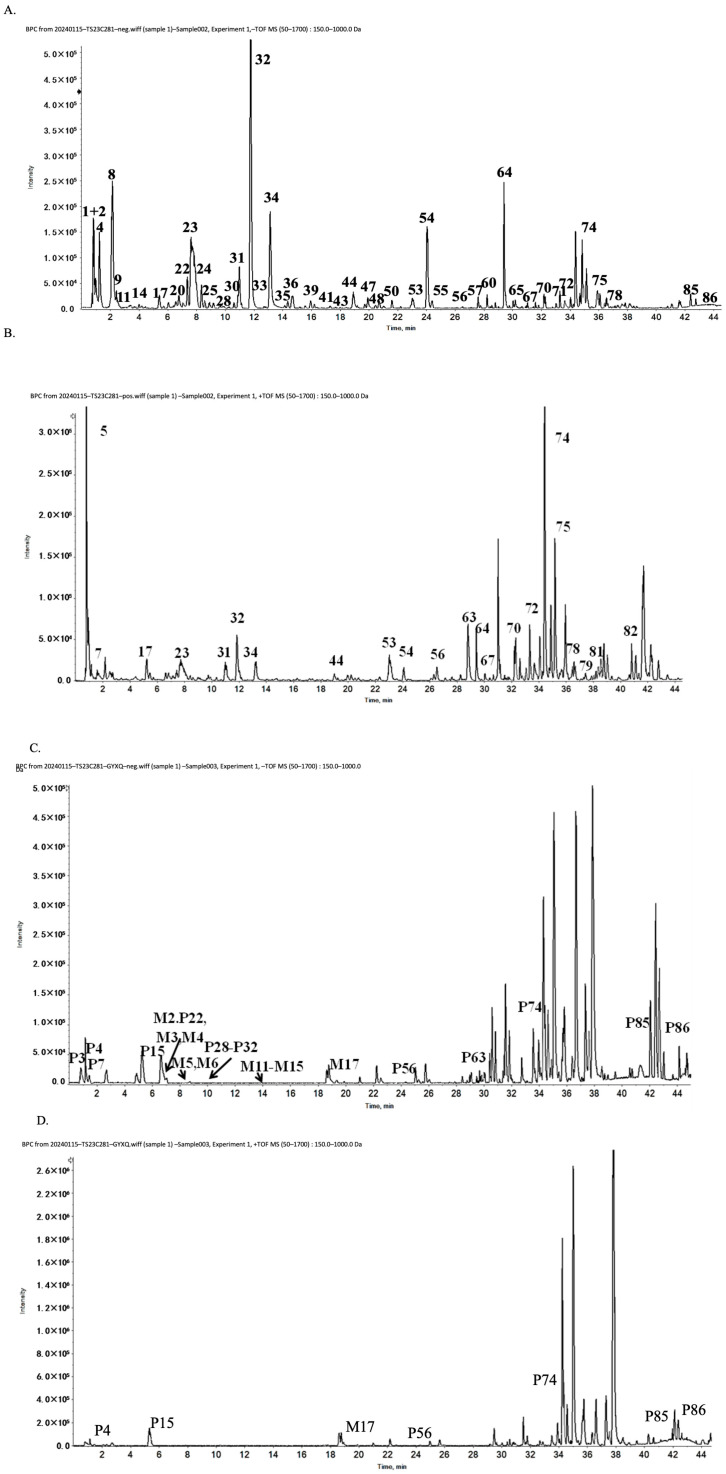

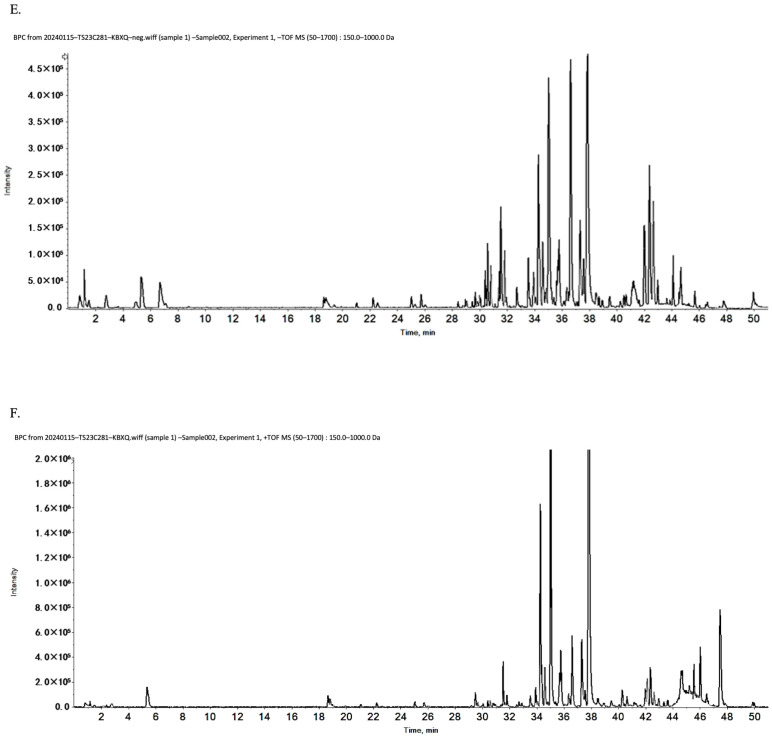
Active constituents of the LDP preparation and LDP-medicated serum identified by UPLC-MS. (**A**,**B**) Base peak ion chromatograms of LDP compound samples in negative and positive ion modes. (**C**,**D**) Base peak ion chromatograms of LDP-medicated serum samples in negative and positive ion modes. (**E**,**F**) Base peak ion chromatograms of blank serum samples in negative and positive ion modes (P, prototype constituents; M, metabolites).

The compound samples were analyzed by UPLC-Q-TOF/MS for qualitative chemical profiling rather than quantitative determination of compound abundance. These data were therefore used to identify the major detectable constituents of the LDP preparation and to provide chemical characterization information for reproducibility, not to compare constituent concentrations or establish concentration–effect relationships. A total of 86 compounds were identified in the LDP preparation (Table 1 and Figure 3), including monoterpene glycosides, iridoid glycosides, phenylethanol glycosides, sesquiterpenes, organic acids, triterpenes, and unsaturated fatty acids. Based on comparative analysis using publicly available chemical composition databases (PubChem and TCMSP), these compounds were assigned to their botanical sources: 23 to Rehmannia (SDH), 24 to Cornus officinalis (SZY), 31 to Cortex Moutan (MDP), 3 to Poria (FL), 8 to Dioscorea opposita (SY), and 13 to Alisma orientalis (ZX).

In addition, 34 LDP-related compounds were detected in serum after administration (Table 2 and Figure 3), including 17 prototype compounds and 17 metabolites involving phase I and phase II metabolic pathways. These serum data provide preliminary information on systemic exposure after LDP administration. Because the present analysis was qualitative, these findings are interpreted as exposure-related chemical evidence rather than as a basis for comparing the abundance or individual efficacy of specific constituents.

### 2.4. LDP Treatment Was Associated with Gut Microbiota Remodeling in PF Mice

**Figure 4:**
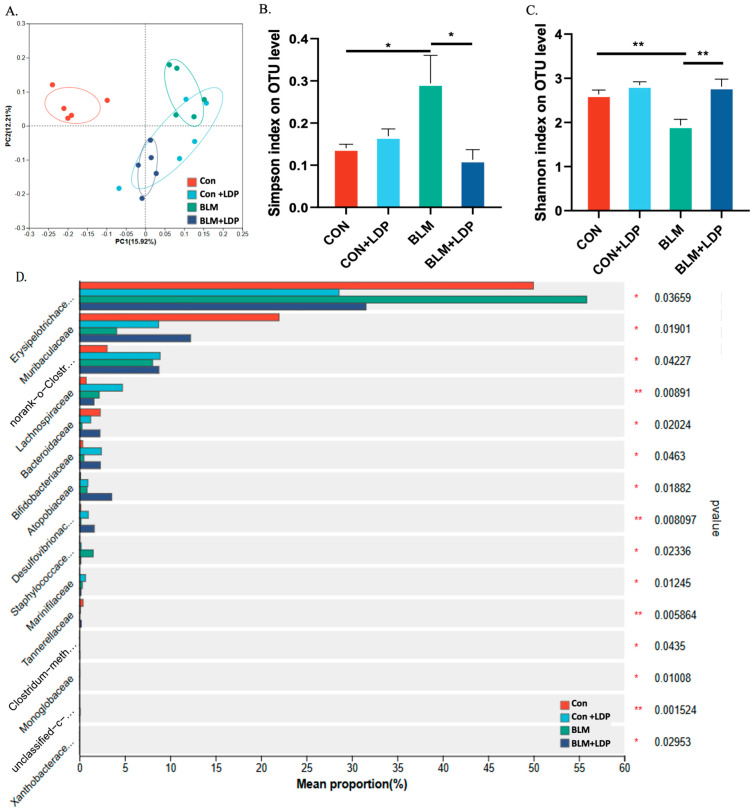
LDP altered gut microbiota structure in mice with BLM-induced pulmonary fibrosis. (**A**) Differences among the CON, CON+LDP, BLM, and BLM+LDP groups were tested by paired PERMANOVA (*n* = 5 per group; R = 0.38, *p* = 0.004). (**B**,**C**) LDP corrected the BLM-induced changes in the Simpson and Shannon indices. (**D**) Comparison of fecal microbiota at the phylum level among control, CON+LDP, BLM, and BLM+LDP mice. * *p* < 0.05, ** *p* < 0.01.

To investigate whether LDP treatment was associated with gut microbiota remodeling, we collected fecal samples before and after treatment for 16S rRNA analysis. Both BLM and LDP were associated with changes in gut microbiota composition (Figure 4A and Figure S1B,C), and LDP significantly improved the BLM-induced reduction in microbial richness (Figure 4B,C). One-way ANOVA showed that BLM increased Erysipelotrichaceae and Lachnospiraceae while reducing Muribaculaceae and Bacteroidaceae (Figure 4D). To further interpret the possible biological relevance of these shifts, we referred to previous studies, which indicated that increases in Erysipelotrichaceae and Lachnospiraceae are associated with inflammatory or fibrotic phenotypes, whereas Muribaculaceae and Bacteroidaceae have been associated with more favorable outcomes in fibrosis-related settings (Lan et al., 2024 [32]; Li et al., 2024 [33]; Su et al., 2024 [34]; Wu et al., 2024 [35]; Ren et al., 2024 [36]).

In addition, LDP was associated with increased abundance of Bifidobacteriaceae and Desulfovibrio, taxa that have shown protective associations in some disease contexts (Sun et al., 2019 [37]). Previous studies suggest that Desulfovibrio is associated with greater susceptibility to pulmonary injury in lung injury models, indicating a potential role in gut–lung axis-related inflammatory regulation, whereas Bifidobacterium abundance has been reported to decrease in patients with PF (Ni et al., 2025 [38]). However, the functional significance of specific taxa, such as Lachnospiraceae and Desulfovibrio, may vary across disease models and host contexts; therefore, these findings are interpreted as treatment-associated microbial alterations rather than definitive evidence of fixed beneficial or harmful roles. Taken together, these data indicate that LDP treatment was accompanied by partial restoration of gut microbial richness and shifts in taxa previously linked to inflammatory or fibrotic phenotypes.

To further examine the transferability of LDP-associated microbiota changes, we performed fecal microbiota transplantation experiments (Figure S3A–F). Fecal transplantation from donor mice significantly altered the microbial structure of recipient mice (Figure S3D). In recipients that underwent both antibiotic pretreatment and BLM modeling, feces from LDP-treated donors improved microbial richness relative to BLM recipients (Figure S3E,F). However, despite altering the microbial structure of recipient mice, FMT produced only limited phenotypic benefit, suggesting that LDP-associated microbiota changes alone were insufficient to transfer the full protective phenotype under the present experimental conditions.

### 2.5. VSL#3 Partially Attenuates PF-Related Changes in Mice

**Figure 5:**
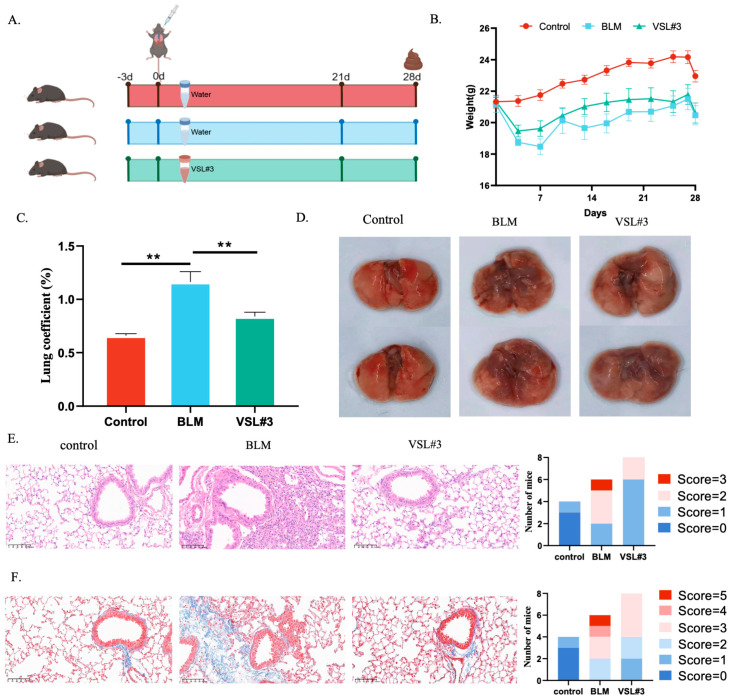
VSL#3 exerted partial therapeutic effects against bleomycin-induced pulmonary fibrosis. (**A**) Mice were divided into three groups, including one normal group and two bleomycin-induced groups receiving water or VSL#3 orally by gavage daily for 28 days. (**B**) VSL#3 restored body weight in mice with PF (*n* = 8 or 6). (**C**,**D**) VSL#3 reduced the lung coefficient and ameliorated gross lung injury in PF mice. (**E**,**F**) Representative HE and Masson staining images (*n* = 8 or 6). Scale bar: 100 μm. HE and Masson staining were evaluated using the Szapiel and Ashcroft methods. ** *p* < 0.01.

VSL#3 was used as a defined probiotic intervention to examine whether microbiota-related modulation could partially affect the PF phenotype. In the VSL#3 study (Figure 5A), intratracheal BLM caused weight loss, elevation of the lung mass index, and grossly visible fibrotic injury, all of which were reversed by VSL#3 treatment (Figure 5B–D). HE and Masson staining showed obvious alveolar damage, alveolar septal edema, alveolar wall thickening, inflammatory cell infiltration, and aggravated interstitial fibrosis in the BLM group (Figure 5E,F), whereas these changes were alleviated by VSL#3. These results indicate that VSL#3 partially attenuated inflammation and fibrosis in this model. Together with the microbiota changes observed after LDP treatment, these findings support the possibility that probiotic-associated microbiota remodeling contributes to protection, although specific microbial mediators and causality remain unresolved.

### 2.6. The Possible Targets and Pathways of LDP Using RNA-Seq

**Figure 6:**
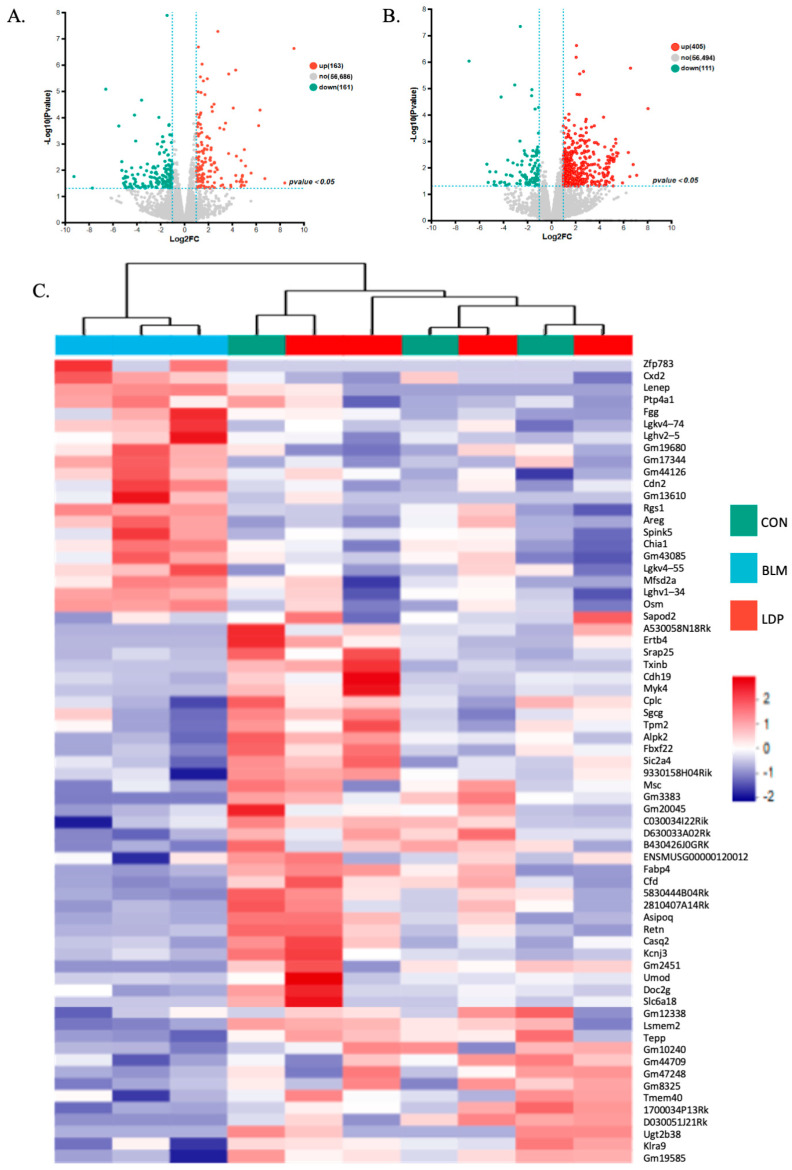

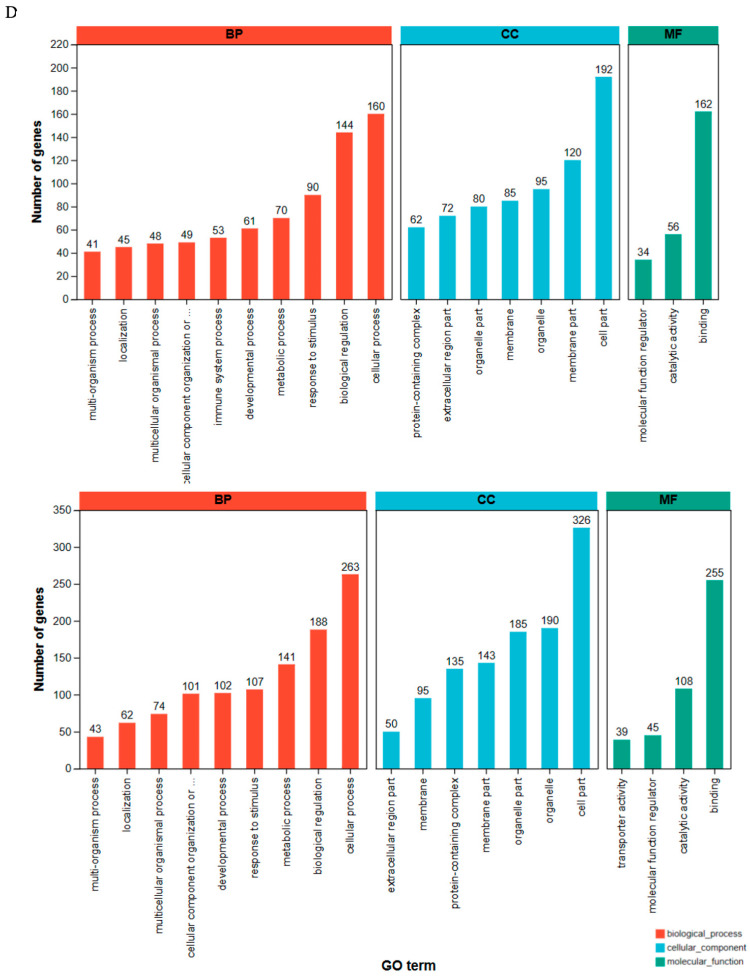
LDP affected gene expression and pathway activity in BLM-induced PF mice. (**A**,**B**) Differentially expressed genes between groups ((**A**), BLM vs. CON; (**B**), LDP vs. BLM), including upregulated and downregulated genes (*p* < 0.05, FC ≥ 2 or FC ≤ 0.5). (**C**) Heatmap of gene expression in the CON, BLM, and LDP groups. (**D**) GO enrichment pathways for BLM vs. CON and LDP vs. BLM.

RNA-seq analysis of lung tissue from the LDP study (Figure 6A–C and Figure S2A,B) showed that BLM altered the expression of 324 genes, including 163 upregulated and 161 downregulated genes. LDP treatment was associated with altered expression of 516 genes in BLM-treated lungs, including 405 upregulated and 111 downregulated genes. Sixty-nine genes overlapped between the two comparisons. Among these, 22 genes upregulated by BLM were reversed by LDP, and 46 genes downregulated by BLM were also reversed by LDP, suggesting restoration toward baseline expression. Heatmap clustering showed clear mRNA expression differences among the control, BLM, and LDP-treated groups (Figure 6C), suggesting broad transcriptomic remodeling after LDP treatment.

GO enrichment analysis (Figure 6D) indicated that the transcriptomic changes associated with LDP treatment were mainly related to metabolic regulation, cellular growth, and injury-response processes.

**Figure 7:**
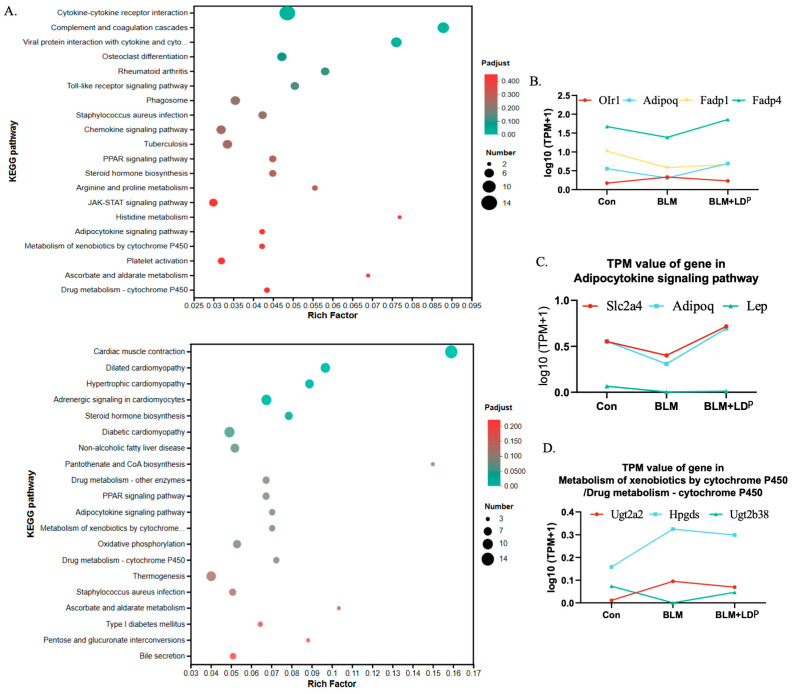
LDP affected genes in related pathways. (**A**) KEGG enrichment pathways for BLM vs. CON and LDP vs BLM. (**B**–**D**) Expression of genes in KEGG pathways in each group.

A combined analysis of the top 20 KEGG pathways enriched after BLM induction and LDP treatment (Figure 7A) identified seven overlapping pathways, suggesting that they may participate in the LDP-associated response to BLM-induced PF. To facilitate interpretation of the enrichment results, we compared the identified pathways with previous studies on fibrosis biology and microbiota-related host responses. Based on this comparison, the PPAR signaling pathway, adipokine signaling pathway, xenobiotic metabolism by cytochrome P450, and drug metabolism via cytochrome P450 were considered potentially relevant to fibrosis biology and have also been reported to be linked to the intestinal microbiota. For example, intestinal microbes can influence PPAR signaling through their metabolites and thereby affect organ fibrosis (Byndloss et al., 2017 [39]; Wu et al., 2024 [35]; Wu et al., 2023 [40]), as well as CYP450 activity and adipokine regulation (Su et al., 2024 [34]; Al-Muzafar & Amin, 2017 [41]; Keskitalo et al., 2018 [42]; Sun et al., 2019 [37]). We further examined genes within these pathways and found that several BLM-induced changes were reversed by LDP (Figure 7B–D). In addition to these pathways, Staphylococcus aureus infection and steroid hormone biosynthesis were also enriched and may be relevant to LDP-associated effects. Gene set enrichment analysis suggested that LDP treatment exhibited upregulation of antimicrobial-related signatures, such as ALPHA_DEFENSINS (Xu et al., 2021 [43]) (Figure S2C), and decreased pro-inflammatory and EMT-related pathways, such as MYD88_INDEPENDENT_TLR4_CASCADE (Tang et al., 2023 [44]) and SEMAPHORIN_INTERACTIONS (Peng et al., 2023 [45]) (Figure S2F,G). In addition, ATP generation-related pathways such as RESPIRATORY_ELECTRON_TRANSPORT and COMPLEX_1_BIOGENESIS, were upregulated (Figure S2D,E), whereas tumor-related signaling, such as SIGNALING_BY_NTRK1_TRKA, was downregulated (Figure S2H). Overall, these transcriptomic data provide mechanistic hypotheses for future validation rather than protein-level confirmation of a single pathway. In particular, PPAR signaling should be interpreted as a priority candidate pathway rather than a confirmed mechanism, because PPARγ and its downstream targets were not validated at the protein level in this study. Although this study did not perform a formal microbiome–transcriptome integration analysis, the two omics datasets showed consistent biological themes. LDP-associated gut microbiota remodeling was accompanied by lung transcriptional changes related to inflammation, fibrosis, metabolic regulation, and antimicrobial defense, providing complementary support for the proposed microbiome–host response framework.

## 3. Discussion

LDP is traditionally used for disorders associated with kidney-yin deficiency, and modern studies have suggested anti-inflammatory and anti-fibrotic activities in other disease settings (Song et al., 2022 [46]; Pan et al., 2023 [16]). In the present study, we extended these observations to a bleomycin-induced PF model and found that LDP attenuated early lung injury and fibrosis-related readouts. In light of the traditional concept of lung–kidney interaction and the growing recognition of multi-organ signaling in fibrosis, these findings provide preclinical evidence that LDP may attenuate early bleomycin-induced PF-related injury in mice.

In the bleomycin-induced PF model, we assessed lung pathology, hydroxyproline content, and inflammatory markers as key indicators of early fibrotic injury (Raghu et al., 2022 [3]). LDP improved histopathology and reduced inflammation- and fibrosis-related indices, supporting a protective effect during the transition from inflammation to fibrosis. The anti-fibrotic effect of LDP was evaluated mainly through lung pathology, lung index, HYP, α-SMA, and inflammatory cytokines, whereas UPLC-Q-TOF/MS was used for qualitative chemical characterization rather than constituent-level dose–effect analysis.

Because gut microbiota has increasingly been implicated in respiratory disease through the gut–lung axis (Shi et al., 2023 [47]), we further examined whether microbiota remodeling was associated with LDP treatment. LDP was associated with altered gut microbial diversity and community structure, and VSL#3 partially reproduced the protective phenotype in PF mice, supporting a possible contribution of microbiota-related changes to the observed protection. However, microbiota sampling was performed only at a terminal time point; so, the present data support association rather than causality. Therefore, the current data cannot determine whether gut microbiota remodeling precedes, accompanies, or follows the attenuation of fibrotic injury. In addition, LDP-containing serum showed only limited inhibition of TGF-β1-induced EMT in A549 cells (Figure S4A–E), despite clear in vivo efficacy. This discrepancy suggests that LDP-mediated protection may depend on systemic host responses, microbiota-related effects, or both, rather than on a simple direct cellular effect of serum-exposed compounds alone. VSL#3 produced a partial protective effect in PF mice, whereas FMT from LDP-treated donors showed only limited phenotypic benefit. The partial and limited efficacy of FMT suggests that microbiota transfer alone may not fully account for the LDP-associated protective phenotype. These two experiments were therefore interpreted as complementary microbiota-related tests: FMT evaluated the transferability of LDP-associated microbiota, whereas VSL#3 examined whether a defined probiotic intervention could partially affect the PF phenotype. Together with previous evidence that gut microbiota can influence bleomycin-induced lung injury and fibrotic outcomes (Yoon et al., 2022 [48]), these results suggest a possible microbiota-related contribution to LDP-associated protection, while also indicating that microbiota transfer alone was insufficient to reproduce the full effect. Building on the established knowledge that drug treatment can modulate gut microbiota, the present study further examined this issue in the specific setting of bleomycin-induced PF. Rather than attributing the therapeutic effect to microbiota changes alone, our findings suggest that LDP-associated protection may involve the combined participation of direct drug effects, microbiota-related modulation, and host responses.

These microbiota-related observations also provide a context for interpreting the transcriptomic findings described below, as the microbial and host transcriptional changes may represent related layers of the same LDP-associated protective response.

To further explore the mechanism of LDP in PF, we performed transcriptome sequencing and KEGG enrichment analysis to identify pathways associated with both bleomycin induction and LDP treatment. Among the enriched transcriptomic pathways, the PPAR signaling pathway appears to be the most relevant candidate in this study. PPARγ activation has been reported to inhibit TGF-β/Smad signaling, extracellular matrix accumulation, and EMT in fibrotic settings (Kökény et al., 2021 [49]), and components of Rehmannia-containing formulas have also been linked to the regulation of PPAR-γ signaling (Zhang et al., 2022 [50]). Meanwhile, the bacterial families altered by LDP and the transcriptomic pathways identified here may be considered co-varying observations (Shi et al., 2025 [51]; Senavonge et al., 2025 [52]). Other enriched pathways, including adipokine-related signaling, xenobiotic metabolism, and CYP-related metabolic processes, may provide additional but secondary clues to LDP-associated host responses (Dileepan et al., 2019 [53]; Guan 2021 [54]; Lau et al., 2013 [55]; Stoilov et al., 2006 [56]). Therefore, RNA-seq in this study was used primarily as a hypothesis-generating approach to prioritize candidate host-response pathways, rather than as definitive pathway validation. Among these pathways, PPAR signaling was prioritized because of its reported links with fibrosis, EMT regulation, metabolic homeostasis, and microbiota-derived signaling. However, these RNA-seq findings still require further validation, with the PPAR signaling pathway representing a priority for future protein-level verification.

Although no formal cross-omics integration analysis was performed, the microbiome and transcriptomic results showed consistency in key biological themes. In addition, 16S rRNA sequencing mainly reflected changes in microbial composition. Future functional prediction, metagenomic analysis, or metabolomic profiling would help further clarify how these microbial changes may be linked to host fibrotic pathways. After LDP intervention, the intestinal microbiota was reshaped, suggesting partial correction of PF-related microecological imbalance, while lung transcriptomics showed downregulation of inflammation- and EMT-related pathways, together with changes in pathways related to metabolic regulation, host defense, and energy metabolism. Together, these findings support a biologically consistent pattern of microbiota alteration accompanied by reduced host inflammatory and fibrotic signaling. However, this finding reflects biological consistency rather than formal causal verification.

The present findings suggest that the protective effect of LDP is unlikely to be mediated by a single mechanism, but rather involves three related levels. First, LDP treatment itself may provide a direct pharmacological basis for protection, as supported by improvements in inflammatory and fibrotic phenotypes and by the reported activities of several known constituents. However, the limited inhibition of EMT by LDP-containing serum in vitro suggests that the in vivo efficacy of LDP cannot be explained solely by direct cellular effects. Second, LDP-associated protection may be linked to gut microbiota remodeling, as indicated by altered microbial structure after treatment and partial phenotypic reproduction by VSL#3. Third, lung transcriptomic analysis suggests coordinated host responses involving inflammation-, EMT-, metabolism-, and defense-related pathways, with PPAR signaling representing a priority candidate for future validation. Taken together, these phenotypic, microbiota-related, chemical, in vitro, and transcriptomic findings support a convergent model in which direct drug exposure, microbiota remodeling, and host responses jointly contribute to the attenuation of early fibrotic injury, although direct causality and targeted pathway validation remain unresolved.

Several limitations should be acknowledged. First, the microbiome analysis was cross-sectional rather than longitudinal, which limits causal interpretation of microbiota changes during PF progression or remission. Second, the UPLC-Q-TOF/MS analysis was qualitative and did not define the abundance or concentration–effect relationships of individual LDP-derived compounds. Targeted quantitative analysis and metabolite profiling are therefore needed to identify the key active effectors in future studies. Third, the transcriptomic findings were not validated at the protein level and should therefore be interpreted as preliminary mechanistic insights. Finally, all efficacy conclusions were derived from the bleomycin model, which mainly reflects inflammatory and early fibrotic features and may not fully represent the heterogeneity of PF. In addition, the gut microbiota changes observed in mice may not be directly extrapolated to humans because of inherent interspecies differences in microbiota composition. Future studies should combine longitudinal microbiome profiling, targeted mechanistic validation, protein-level verification of prioritized pathways, and additional PF models to strengthen generalizability.

## 4. Materials and Methods

### 4.1. Chemicals and Reagents

Liuweidihuang pill (LDP; batch number: 2220723; national drug approval number: Z41022128) was purchased from Zhongjing Wanxi Pharmaceutical Co., Ltd. (Nanyang, China). Pirfenidone (PFD; batch number: 20210511; national drug approval number: H20133376) was obtained from Gyre Therapeutics, Inc. (San Diego, CA, USA). Bleomycin (BLM; CAS: 9041-93-4) was purchased from Shanghai Macklin Biochemical Technology Co., Ltd. (Shanghai, China). Transforming growth factor-β1 (TGF-β1; HEK293-derived; catalog number: 100-21; lot number: 0922209-1) was obtained from PeproTech Inc. (Cranbury, NJ, USA). VSL#3 (lot number: G5H019) was purchased from VSL Pharmaceuticals, Inc. (Fort Lauderdale, FL, USA).

### 4.2. Confirmatory Experiments for Efficacy Validation

#### 4.2.1. LDP Treatment in PF Mice

Adult male C57BL/6J mice (20–22 g, 7–8 weeks old) were obtained from Shanghai Jihui Experimental Animal Breeding Co., Ltd. (Shanghai, China) or Shanghai Slake Experimental Animal Co., Ltd. (Shanghai, China). Adult male SD rats (180–220 g, 6–8 weeks old) were obtained from Shanghai Slake Experimental Animal Co., Ltd. Animals were housed under specific-pathogen-free conditions at the Laboratory Animal Center of Shanghai University of Traditional Chinese Medicine (certificate no. SYXK (Hu) 2020-0009) under a 12 h light/dark cycle at 20–22 °C and 45 ± 5% humidity. All animal experiments were reviewed and approved by the Animal Care and Use Committee of Shanghai University of Traditional Chinese Medicine (approval numbers: PZSHUTCM220124010; PZSHUTCM2302110004; PZSHUTCM2306070002), and were conducted in accordance with NIH guidelines. The sample size was determined based on previous studies (Yu et al., 2022 [57]). Given the nature of the interventions, experimenters administering drugs and handling animals were not blinded to group allocation, but sample collection and histological assessment were performed in a blinded manner. Lung sections were coded by an independent researcher and evaluated by two blinded pathologists. Although treatment administration was not blinded, subsequent blinded sampling, coding, and pathological assessment helped reduce potential bias in result interpretation. Animals were anesthetized with 2% pentobarbital sodium (40 mg/kg, intraperitoneally) before sample collection.

For the LDP intervention study, C57BL/6J mice (*n* = 70) were stratified by body weight and randomly assigned to seven groups (*n* = 10/group): CON, CON+LDP, BLM, BLM+PFD, BLM+LDP-L, BLM+LDP-M, and BLM+LDP-H. The model and treatment groups received a single intratracheal instillation of 5 mg/kg BLM. The PFD group received 300 mg/kg/day PFD by gavage. Based on clinical dose conversion, the mouse-equivalent dose of LDP was estimated to be approximately 600 mg/kg/day. Based on dose ranges reported in previous LDP-related animal studies, 600 mg/kg/day was used as the equivalent dose, and 1200 mg/kg/day was used as the high dose to expand the observation range (Perry et al., 2012 [58]), while the low dose was about 300 mg/kg/day. Because no systematic pharmacokinetic analysis was performed in this study, the rationale underlying these dose conversions requires further investigation. The three LDP groups received 300, 600, or 1200 mg/kg/day by gavage after modeling. Body weight was recorded every 3 days for 4 weeks (July–August 2022). Pulmonary pathology, lung index, α-SMA, HYP, and inflammatory factors were subsequently assessed.

#### 4.2.2. Hematoxylin–Eosin Staining (HE) and Masson Staining

The left lung was fixed by tracheal perfusion with 4% paraformaldehyde and embedded in paraffin. Sections were baked, dewaxed in xylene, and rehydrated through graded ethanol. HE staining was performed with hematoxylin and eosin, and Masson staining was performed using the corresponding kit reagents. Histological injury and fibrosis were evaluated in a blinded manner by two experienced pathologists using the Szapiel and Ashcroft methods, respectively.

#### 4.2.3. Immunofluorescence

Tissue sections underwent antigen retrieval, endogenous peroxidase blocking with 3% methanol peroxide, and incubation with blocking solution. Sections were then incubated with primary antibodies overnight at 4 °C, followed by washing, secondary antibody incubation for 1.5 h at room temperature, DAPI staining (50 μg/mL), and mounting with xylene.

#### 4.2.4. Enzyme-Linked Immunosorbent Assay

Fresh mouse blood was left at room temperature for 15 min and centrifuged to collect serum. ELISA kits (Jiangsu Meimian Industrial Co., Ltd., Yancheng, China) were used to measure interleukin-6 (IL-6), interleukin-1β (IL-1β), and tumor necrosis factor-α (TNF-α). Absorbance was read at 450 nm.

#### 4.2.5. Hydroxyproline Assay

Serum HYP was measured using a commercial assay kit (Nanjing Jiancheng Biotechnology Research Institute Co., Ltd., Nanjing, China) according to the manufacturer’s instructions. Absorbance was read at 450 nm.

#### 4.2.6. Ultra-Performance Liquid Chromatography-Mass Spectrometry (UPLC-MS)

Chemicals: All reagents and solvents were MS-grade. Acetonitrile (LOT211022), methanol (LOT21587), and formic acid (LOT2100122) were purchased from Thermo Fisher Scientific. Water (GB21038) was obtained from Guangzhou Wastson’s Food & Beverage Co., Ltd. (Guangzhou, China).

Sample Preparation: Briefly, 0.5 g of powdered compound sample that were passed through a No. 4 sieve was mixed with 10 mL of 70% methanol in a sealed flask and subjected to ultrasonication at 100 W for 30 min. After cooling, solvent loss was replenished with 50% methanol. A 2ml aliquot was centrifuged and the supernatant was collected. Serum samples were thawed at 4 °C, mixed with three volumes of methanol, centrifuged, and the supernatant was concentrated to dryness, stored at −20 °C, and reconstituted in 200 μL of 50% methanol prior to analysis.

UPLC-MS Analysis: Compound and serum samples were analyzed using a UPLC-Q-TOF/MS system (Shimadzu, Japan/SCIEX, Shanghai, China) equipped with a Waters ACQUITY UPLC HSS T3 C18 column (1.7 μm, 2.1 × 100 mm). Mobile phase A was 0.1% formic acid in water and mobile phase B was acetonitrile. The gradient was 3% B at 0 min, 8% at 5 min, 13% at 15 min, 20% at 25 min, 50% at 30 min, 75% at 40 min, and 95% at 45 min. The flow rate was 0.3 mL/min, the column temperature was 30 °C, and the injection volume was 2 μL. MS data were acquired in both positive and negative ESI modes; detailed parameters are shown in Table 3.

### 4.3. Exploratory Experiments for Mechanistic Investigations

#### 4.3.1. Animal Experiments

Animal species, housing conditions, randomization, and blinding procedures were the same as those described in Section 4.2.1. Two microbiota-related strategies were used in the exploratory experiments: fecal microbiota transplantation (FMT), to assess whether LDP-associated microbiota changes were transferable, and VSL#3 intervention, to test whether a defined probiotic mixture could partially mimic the microbiota-related protective phenotype.

For the FMT study, C57BL/6J mice (*n* = 30) were divided into six groups: CON, BLM, BLM+LDP, R-CON, R-BLM, and R-LDP (*n* = 5 each). Fresh feces (200 mg) from donor mice were collected daily, suspended in 5 mL saline, vortexed for 3 min, and allowed to settle for 2 min. Recipient mice were pretreated with antibiotics and then gavaged once daily on day 5 with 200 μL fecal supernatant. Body weight was recorded daily for 4 weeks (December 2022–January 2023). Outcomes included body weight and gut microbiota diversity.

For the VSL#3 intervention study, C57BL/6J mice (*n* = 24) were divided into CON, BLM, and VSL#3 groups (*n* = 8 each). The BLM groups received a single intratracheal instillation of 5 mg/kg BLM. The VSL#3 group received 2 × 10^8^ CFU VSL#3 by gavage. Body weight was recorded daily for 4 weeks (December 2023–January 2024). Two mice in the BLM group were excluded because they died from asphyxiation during modeling. Outcomes included body weight, lung pathology, and lung index.

For serum collection, SD rats (*n* = 20) were divided into CON and CON+LDP groups (*n* = 10 each). Rats in the LDP group received 2.7 g/kg LDP twice daily for 5 days (April 2024). On day 6, blood was collected from the abdominal aorta under sodium pentobarbital anesthesia (80 mg/kg, intraperitoneally), centrifuged, filtered through a 0.22 μm membrane, and stored at −80 °C for serum constituent analysis and cell experiments.

#### 4.3.2. 16S rRNA Gene Sequencing

Fecal bacterial DNA was extracted using the PF Mag-Bind Stool DNA Kit (Omega Bio-Tek, Norcross, GA, USA) according to the manufacturer’s instructions. DNA quality and concentration were assessed by 1.0% agarose gel electrophoresis and NanoDrop ND-2000 (Thermo Scientific, Waltham, MA, USA). The V3-V4 region of the 16S rRNA gene was amplified with primers ACTCCTACGGGAGGCAGCAG and GGACTACHVGGGTWTCTAAT. PCR products were purified, quantified, pooled equimolarly, and sequenced on the Illumina NextSeq 2000 PE300 platform (Illumina, SD, USA).

Raw reads were demultiplexed, quality-filtered with fastp (v0.19.6), merged with FLASH (v1.2.11), and checked for chimeras. High-quality reads were clustered into OTUs at 97% identity using UCLUST, and representative sequences were selected. Rarefaction curves and Good’s coverage values (>99%) indicated adequate sequencing depth. OTU tables were rarefied to the minimum depth, and abundances were expressed as relative frequencies with Z-score normalization for heatmap analysis.

Alpha diversity was assessed using the Chao1 and Shannon indices. Beta diversity was calculated from Bray–Curtis distances and visualized by PCoA, and group differences were tested by PERMANOVA (*p* < 0.05). Differential taxa were identified using the Kruskal–Wallis test with FDR correction (Q < 0.05), followed by Welch’s *t*-test for post hoc comparisons.

#### 4.3.3. RNA-Seq

Total RNA was extracted from tissue using TRIzol reagent according to the manufacturer’s instructions. RNA purification, reverse transcription, library construction, and sequencing were performed by Shanghai Majorbio Bio-pharm Biotechnology Co., Ltd. (Shanghai, China). Libraries were prepared using Illumina Stranded mRNA Prep (Illumina, SD, USA) with 1 μg total RNA and sequenced on a NovaSeq X Plus platform (Illumina, SD, USA). All samples were processed in a single batch to minimize batch effects. Differential expression analysis was performed using DESeq2 (v1.38.0). Genes with |log_2_ fold change| ≥ 1 (corresponding to fold change ≥ 2 or ≤ 0.5) and raw *p*-value < 0.05 were considered significantly differentially expressed. To address multiple testing in downstream enrichment analyses, we applied the Benjamini–Hochberg (BH) procedure for *p*-value correction. Functional enrichment analyses, including Gene Ontology (GO), Kyoto Encyclopedia of Genes and Genomes (KEGG) pathway, and Gene Set Enrichment Analysis (GSEA), were conducted to identify biological processes and pathways significantly associated with the differentially expressed genes. GO enrichment analysis was performed using Goatools (v0.6.5), KEGG pathway analysis using KOBAS (v2.1.1), and GSEA using the MSigDB database. Enriched terms with BH-adjusted *p* < 0.05 were considered significant.

#### 4.3.4. Cell Experiments

A549 cells were obtained from the Cell Bank of the Chinese Academy of Sciences (Shanghai, China) and cultured in DMEM containing 10% inactivated fetal bovine serum at 37 °C in 5% CO_2_. After 12 h, cells were serum-starved for an additional 12 h and then treated with 10 ng/mL TGF-β1 for 24 h to induce epithelial–mesenchymal transition.

### 4.4. Statistical Analysis

The 16S rRNA and RNA-seq data were analyzed using the Majorbio platform (Shanghai Majorbio Bio-pharm Technology Co., Ltd., Shanghai, China). Graphs were generated with GraphPad Prism 8.0.2. Data were first assessed for normality and homogeneity of variance. One-way or two-way ANOVA was used when parametric assumptions were met; otherwise, appropriate non-parametric tests were applied. In vivo data are presented as mean ± SD, and *p* < 0.05 was considered statistically significant.

## 5. Conclusions

In conclusion, LDP attenuated early bleomycin-induced pulmonary fibrosis and reduced inflammatory responses in mice. These effects were accompanied by gut microbiota remodeling and transcriptomic changes, thereby providing potential mechanistic insights. Thus, the present study extends the understanding of LDP-associated protection beyond efficacy assessment alone. These findings provide preclinical evidence relevant to PF treatment and support further translational evaluation of LDP in future studies. However, the present study does not establish causal microbiota effects, identify the key active effectors, or validate transcriptomic pathways at the protein level. Future studies should therefore prioritize targeted validation of the most relevant candidate pathway, together with longitudinal assessment of microbiota changes and more precise identification of active effectors.

## Figures and Tables

**Table 1:** Main components of compound samples by UPLC-Q-TOF/MS.

No	Retention Time (min)	Adduct Ion	*m*/*z* Actual Value	*m*/*z* Theoretical Value	Molecular Formula	Name	Source
**1**	0.797	[M−H]^−^	341.1078	341.1089	C_12_H_22_O_11_	Sucrose	FL, SDH, MDP, SY
**2**	0.814	[M+FA−H]^−^	549.1682	549.1672	C_18_H_32_O_16_	Maltotriose	SDH, SY
**3**	0.844	[M−H]^−^	191.0566	191.0556	C_7_H_12_O_6_	Quinic acid	SDH
**4**	1.229	[M−H]^−^	191.0204	191.0192	C_6_H_8_O_7_	Citric acid	SDH, SZY, MDP, SY
**5**	1.283	[M+H]^+^	130.0498	130.0504	C_5_H_7_NO_3_	L-Pyroglutamic acid	MDP
**6**	1.361	[M−H]^−^	243.0605	243.0617	C_9_H_12_N_2_O_6_	Uridine	SDH
**7**	1.578	[M+H]^+^	268.1036	268.1046	C_10_H_13_N_5_O_4_	Adenosine	SDH, MDP, SY
**8**	2.13	[M−H]^−^	169.015	169.0142	C_7_H_6_O_5_	Gallic acid	SDH, SZY, MDP
**9**	2.405	[M−H]^−^	361.0796	361.0776	C_14_H_18_O_11_	7-O-galloyl-D-sedoheptulose	SZY
**10**	3.06	[M−H]^−^	493.1212	493.1194	C_19_H_26_O_15_	1′-O-galloylsucrose	MDP
**11**	3.4	[M+FA−H]^−^	731.2273	731.2246	C_27_H_42_O_20_	Rehmannioside D	SDH
**12**	3.616	[M−H]^−^	493.1213	493.1194	C_19_H_26_O_15_	6′-O-galloylsucrose	MDP
**13**	3.652	[M+FA−H]^−^	569.174	569.1718	C_21_H_32_O_15_	Rehmannioside A	SDH
**14**	3.989	[M−H]^−^	153.0198	153.0193	C_7_H_6_O_4_	Protocatechuic acid	SDH, SZY, SY
**15**	5.726	[M+FA−H]^−^	421.1348	421.1346	C_16_H_24_O_10_	Desbenzoylpaeoniflorin	MDP
**16**	6.035	[M−H]^−^	461.1678	461.1659	C_20_H_30_O_12_	Decaffeoyl-acteoside	SDH
**17**	6.384	[M−H]^−^	375.1301	375.1291	C_16_H_24_O_10_	8-epiloganic acid	SDH
**18**	6.482	[M+FA−H]^−^	509.1314	509.1301	C_18_H_24_O_14_	Mudanoside B	MDP
**19**	6.611	[M−H]^−^	527.1413	527.1401	C_23_H_28_O_14_	6′-O-Galloyl desbenzoylpaeoniflorin	MDP
**20**	6.767	[M−H]^−^	567.1945	567.1931	C_23_H_36_O_16_	Cornusdiglycoside G	SZY
**21**	6.809	[M−H]^−^	183.0305	183.0294	C_8_H_8_O_5_	Methyl gallate	MDP
**22**	7.355	[M−H]^−^	375.1296	375.1291	C_16_H_24_O_10_	Loganic acid	SDH, SZY
**23**	7.609	[M+FA−H]^−^	451.1473	451.1457	C_17_H_26_O_11_	Morroniside	SZY
**24**	8.336	[M−H]^−^	495.1523	495.1503	C_23_H_28_O_12_	Oxypaeoniflorin	MDP
**25**	8.592	[M−H]^−^	389.108	389.1089	C_16_H_22_O_11_	Secologanoside	SZY
**26**	8.647	[M−H]^−^	179.0357	179.035	C_9_H_8_O_4_	Caffeic acid	SZY
**27**	8.888	[M−H]^−^	513.0898	513.0886	C_21_H_22_O_15_	1,7-di-O-galloyl-D-sedoheptulose	SZY
**28**	10.48	[M+FA−H]^−^	433.1355	433.1352	C_17_H_24_O_10_	Geniposide	SDH
**29**	10.609	[M+FA−H]^−^	435.1514	435.1508	C_17_H_26_O_10_	β-Dihyddrocornin	SZY
**30**	10.962	[M+FA−H]^−^	433.1367	433.1352	C_17_H_24_O_10_	Verbenalin	SZY
**31**	10.972	[M+FA−H]^−^	403.1256	403.1246	C_16_H_22_O_9_	Sweroside	SZY
**32**	11.763	[M+FA−H] ^−^	435.1512	435.1508	C_17_H_26_O_10_	Loganin	SZY
**33**	11.935	[M−H]^−^	197.0459	197.045	C_9_H_10_O_5_	Ethyl gallate	MDP
**34**	13.127	[M+FA−H]^−^	525.1636	525.1608	C_23_H_28_O_11_	Paeoniflorin	MDP
**35**	14.35	[M+FA−H]^−^	505.159	505.1563	C_20_H_28_O_12_	Paeonolide	MDP
**36**	14.703	[M+FA−H]^−^	505.1581	505.1563	C_20_H_28_O_12_	Apiopaeonoside	MDP
**37**	14.703	[M−H]^−^	647.164	647.1612	C_30_H_32_O_16_	Galloyloxypaeoniflorin	MDP
**38**	15.937	[M−H]^−^	505.1588	505.1563	C_21_H_30_O_14_	Cornuside III	SZY
**39**	16.196	[M−H]^−^	785.2549	785.2504	C_35_H_46_O_20_	Purpureaside C	SDH
**40**	16.38	[M+FA−H]^−^	555.1719	555.1746	C_24_H_30_O_12_	Mudanpioside D	MDP
**41**	17.3	[M+FA−H]^−^	435.2242	435.2236	C_19_H_34_O_8_	Rehmaionoside A	SDH
**42**	17.686	[M−H]^−^	505.157	505.1563	C_21_H_30_O_14_	Cornuside IV	SZY
**43**	18.842	[M−H]^−^	477.0687	477.0675	C_21_H_18_O_13_	Quercetin-3-O-β-D-glucuronide	SZY
**45**	19.182	[M−H]^−^	799.2704	799.2666	C_36_H_48_O_20_	Jionoside A1	SDH
**46**	19.712	[M+FA−H]^−^	525.1636	525.1608	C_23_H_28_O_11_	Albiflorin	MDP
**47**	19.916	[M−H]^−^	631.1681	631.1663	C_30_H_32_O_15_	Galloylpaeoniflorin	MDP
**48**	20.445	[M+FA−H]^−^	479.1784	479.177	C_19_H_30_O_11_	7-O-ethyl-Morroniside	SZY
**49**	20.708	[M+FA−H]^−^	479.1783	479.177	C_19_H_30_O_11_	ethyl-Morroniside isomer	SZY
**50**	21.596	[M−H]^−^	623.2005	623.1976	C_29_H_36_O_15_	Verbascoside	SDH
**51**	22.914	[M−H]^−^	615.1739	615.1714	C_30_H_32_O_14_	Mudanpioside H	MDP
**52**	22.994	[M−H]^−^	813.2841	813.2823	C_37_H_50_O_20_	Jionoside B1	SDH
**53**	23.079	[M−H]^−^	623.2012	623.1976	C_29_H_36_O_15_	Isoaceteoside	SDH
**54**	24.043	[M−H]^−^	541.159	541.1563	C_24_H_30_O_14_	Cornuside	SZY
**55**	24.38	[M+FA−H]^−^	507.1525	507.1508	C_23_H_26_O_10_	Lactiflorin	MDP
**56**	26.515	[M+H]^+^	679.5134	679.5117	C_36_H_66_N_6_O_6_	Cyclic hexaleucine	MDP
**57**	27.587	[M−H]^−^	599.179	599.1765	C_30_H_32_O_13_	Mudanpioside C	MDP
**58**	27.74	[M−H]^−^	629.1908	629.1871	C_31_H_34_O_14_	Mudanpioside J	MDP
**59**	28.154	[M−H]^−^	651.2312	651.2294	C_31_H_40_O_15_	Cistanoside D	SDH
**60**	28.221	[M−H]^−^	599.1802	599.1765	C_30_H_32_O_13_	Benzoyloxypaeoniflorin	MDP
**61**	28.51	[M−H]^−^	629.1894	629.1871	C_31_H_34_O_14_	Mudanpioside B	MDP
**62**	28.545	[M−H]^−^	651.2333	651.2294	C_31_H_40_O_15_	Martynoside	SDH
**63**	28.795	[M+H]^+^	167.0706	167.0708	C_9_H_10_O_3_	Paeonol	MDP
**64**	29.392	[M+FA−H]^−^	629.1894	629.187	C_30_H_32_O_12_	Benzoylpaeoniflorin	MDP
**65**	29.764	[M+FA−H]^−^	629.1892	629.187	C_30_H_32_O_12_	Benzoylalbiflorin	MDP
**66**	30.179	[M−H]^−^	329.2342	329.2333	C_18_H_34_O_5_	Trihydroxyoctadecanoic acid	SDH
**67**	30.653	[M+H]^+^	505.3524	505.3524	C_30_H_48_O_6_	16-oxoalisol A	ZX
**68**	30.897	[M+H]^+^	245.1174	245.1172	C_15_H_16_O_3_	Batatasin III	SY
**69**	31.827	[M−H2O+H]^+^	489.3602	489.3575	C_30_H_50_O_6_	13,17-epoxyalisol A	ZX
**70**	32.295	[M+H]^+^	487.3409	487.3418	C_30_H_46_O_5_	Alisol C	ZX
**71**	33.063	[M−H2O+H]^+^	471.3485	471.3469	C_30_H_48_O_5_	Alisol F	ZX
**72**	33.441	[M+H]^+^	529.3539	529.3524	C_32_H_48_O_6_	Alisol C 23-acetate	ZX
**73**	33.711	[M+H]^+^	469.3334	469.3312	C_30_H_44_O_4_	Alisol L	ZX
**74**	34.839	[M+FA−H]^−^	535.3658	535.364	C_30_H_50_O_5_	Alisol A	ZX
**75**	35.28	[M−H2O+H]^+^	515.3741	515.3731	C_32_H_52_O_6_	Alisol A 23-acetate	ZX
**76**	35.395	[M−H]^−^	483.3122	483.3111	C_30_H_44_O_5_	Poricoic acid B	FL
**77**	36.231	[M−H]^−^	469.3325	469.3323	C_30_H_46_O_4_	16α-Hydroxydehydrotrametenolic acid	FL
**78**	36.449	[M−H2O+H]^+^	455.3535	455.352	C_30_H_48_O_4_	Alisol G	ZX
**79**	36.572	[M+H]^+^	515.375	515.3731	C_32_H_50_O_5_	Alisol B 23-acetate	ZX
**80**	36.671	[M−H2O+H]^+^	455.3536	455.352	C_30_H_48_O_4_	Alisol B	ZX
**81**	37.226	[M−H2O+H]^+^	515.3751	515.3731	C_32_H_52_O_6_	Alisol A 24-acetate	ZX
**82**	40.621	[M+H]^+^	499.3801	499.3782	C_32_H_50_O_4_	11-Deoxy-alisol B 23-acetate	ZX
**83**	41.08	[M−H]^−^	455.3542	455.3531	C_30_H_48_O_3_	Ursolic acid	SZY
**84**	41.613	[M−H]^−^	455.3539	455.3531	C_30_H_48_O_3_	Betulinic acid	SZY
**85**	42.751	[M−H]^−^	279.2335	279.233	C_18_H_32_O_2_	Linoleic acid	SZY, SY
**86**	44.205	[M−H]^−^	255.2343	255.233	C_16_H_32_O_2_	Palmitic acid	SZY, SY

**Table 2:** Main components of compound administration serum samples by UPLC-Q-TOF/MS.

No.	Retention Time (min)	Adduct Ion	*m*/*z* Actual Value	*m*/*z* Theoretical Value	Molecular Formula	Name
**P3**	0.844	[M−H]^−^	191.0569	191.0556	C_7_H_12_O_6_	Quinic acid
**P4**	1.218	[M−H]^−^	191.0204	191.0192	C_6_H_8_O_7_	Citric acid
**P6**	1.334	[M−H]^−^	243.062	243.0617	C_9_H_12_N_2_O_6_	Uridine
**P7**	1.562	[M+H]^+^	268.1032	268.1046	C_10_H_13_N_5_O_4_	Adenosine
**P15**	5.577	[M+FA−H]^−^	421.1346	421.1346	C_16_H_24_O_10_	Desbenzoylpaeoniflorin
**M1**	5.851	[M−H]^−^	183.0306	183.0299	C_8_H_8_O_5_	Gallic acid+Methylation
**M2**	7.187	[M−H]^−^	375.1301	375.1297	C_16_H_24_O_10_	desbenzoyPaeoniflorin/Loganin+ Demethylation
**P22**	7.23	[M−H]^−^	375.1305	375.1291	C_16_H_24_O_10_	Loganic acid
**M3**	7.282	[M+FA−H]^−^	435.1523	435.1508	C_17_H_26_O_10_	Morroniside+dehydroxylation
**P23**	7.609	[M+FA−H]−	451.1472	451.1457	C_17_H_26_O_11_	Morroniside
**M4**	7.907	[M−H]^−^	373.0732	373.0776	C_15_H_18_O_11_	Gallic acid +glucuronidation
**M5**	8.12	[M−H]^−^	327.0714	327.0722	C_14_H_16_O_9_	Paeonol+Demethylation+Glucuronidation
**M6**	9.043	[M−H]^−^	307.0482	307.0493	C_11_H_16_O_8_S	Loganin+deglycosylation+sulfation
**P28**	10.304	[M+FA−H]^−^	433.1344	433.1352	C_17_H_24_O_10_	Geniposide
**P29**	10.45	[M+FA−H]^−^	435.1521	435.1508	C_17_H_26_O_10_	β-Dihyddrocornin
**M7**	10.613	[M−H]^−^	230.9978	230.9969	C_8_H_8_O_6_S	Resacetophenone+Sulfation
**M8**	10.654	[M−H]^−^	151.0408	151.0401	C_8_H_8_O_3_	Resacetophenone
**P30**	10.807	[M+FA−H]^−^	433.1365	433.1352	C_17_H_24_O_10_	Verbenalin
**M9**	10.827	[M−H]^−^	403.1282	403.1246	C_17_H_24_O_11_	Morroniside+deglycosylation+dehydration+hydrogenation+glucuronidation
**P31**	10.831	[M+FA−H]^−^	403.1266	403.1246	C_16_H_22_O_9_	Sweroside
**P32**	11.593	[M+FA−H]^−^	435.1508	435.1508	C_17_H_26_O_10_	Loganin
**M10**	11.647	[M−H]^−^	401.1105	401.1089	C_17_H_22_O_11_	Morroniside+deglycosylation+dehydration+glucuronidation
**M11**	11.656	[M−H]^−^	341.0902	341.0878	C_15_H_18_O_9_	Paeonol+Glucuronidation
**M12**	11.822	[M−H]^−^	261.0076	261.0074	C_9_H_10_O_7_S	Paeonol+Hydroxylation+Sulfation
**M13**	12.459	[M−H]^−^	357.0866	357.0827	C_15_H_18_O_10_	Paeonol+Hydroxylation+Glucuronidation
**M14**	12.459	[M−H]^−^	357.0839	357.0822	C_15_H_18_O_10_	Caffeicacid+hydrogenation+glucuronidation
**M15**	12.605	[M−H]^−^	403.125	403.1246	C_17_H_24_O_11_	Morroniside+deglycosylation+dehydration+hydrogenation+glucuronidation
**M16**	13.435	[M−H]^−^	245.0125	245.0123	C_9_H_10_O_6_S	Paeonol+Sulfation
**M17**	18.821	[M−H]^−^	343.1423	343.1398	C_16_H_24_O_8_	Mudanpioside F
**P56**	24.198	[M+H]^+^	679.5144	679.5117	C_36_H_66_N_6_O_6_	Cyclic hexaleucine
**P63**	28.848	[M+H]^+^	167.0691	167.0708	C_9_H_10_O_3_	Paeonol
**P74**	34.752	[M+FA−H]^−^	535.3693	535.364	C_30_H_50_O_5_	Alisol A
**P85**	42.691	[M−H]^−^	279.2337	279.233	C_18_H_32_O_2_	Linoleic acid
**P86**	44.128	[M−H]^−^	255.2343	255.233	C_16_H_32_O_2_	Palmitic acid

**Table 3:** Mass parameters.

MS Parameters	Parameter Values	MS/MS Parameters	Parameter Values
TOF mass range	100~1500	MS/MS mass range	50~1250
Ion Source Gas 1 (psi)	50	Declustering Potential (V)	100
Ion Source Gas 2(psi)	50	Collision Energy (eV)	±40
Curtain Gas (psi)	30	Collision Energy Spread (eV)	20
Ion Spray Voltage Floating (V)	−0.9	Ion Release Delay (ms)	30
Ion Source Temperature (°C)	450	Ion Release Width (ms)	15
Declustering Potential (V)	100		
Collision Energy (eV)	10		

## Data Availability

The original contributions presented in this study are included in the article/Supplementary Material. Further inquiries can be directed to the corresponding author.

## Supplementary Materials

The following supporting information can be downloaded at: https://www.mdpi.com/article/10.3390/ph19050762/s1.

## Abbreviations

α-SMA	α-smooth muscle actin	MS	Mass spectrometry
ADRs	adverse drug reactions	PF	Pulmonary fibrosis
BLM	Bleomycin	PFD	Pirfenidone
EMT	epithelial–mesenchymal transition	SDH	Rehmannia
FL	tuckahoe	SY	Yam
FMT	fecal microbiota transplantation	SZY	Cornel
HE	Hematoxylin–eosin staining	TCM	Traditional Chinese Medicine
HYP	hydroxyproline	TGF-β1	Transforming Growth Factor-beta 1
IL-1β	interleukin-1β	TNF-α	tumor necrosis factor-α
IL-6	Interleukin-6	UPLC-MS	Ultra-Performance Liquid Chromatography-Mass Spectrometry
LDP	Liuweidihuang Pill	ZX	Alisma orientalis
MDP	Cortex Moutan		
