# Supplementary figures and images for: Liuweidihuang Pill Attenuates Early Bleomycin-Induced Pulmonary Fibrosis in Mice and Is Associated with Gut Microbiome
Source: Pharmaceuticals (Basel). 2026 May 13;19(5):762. doi: 10.3390/ph19050762 (PMC13210809; doi:10.3390/ph19050762)

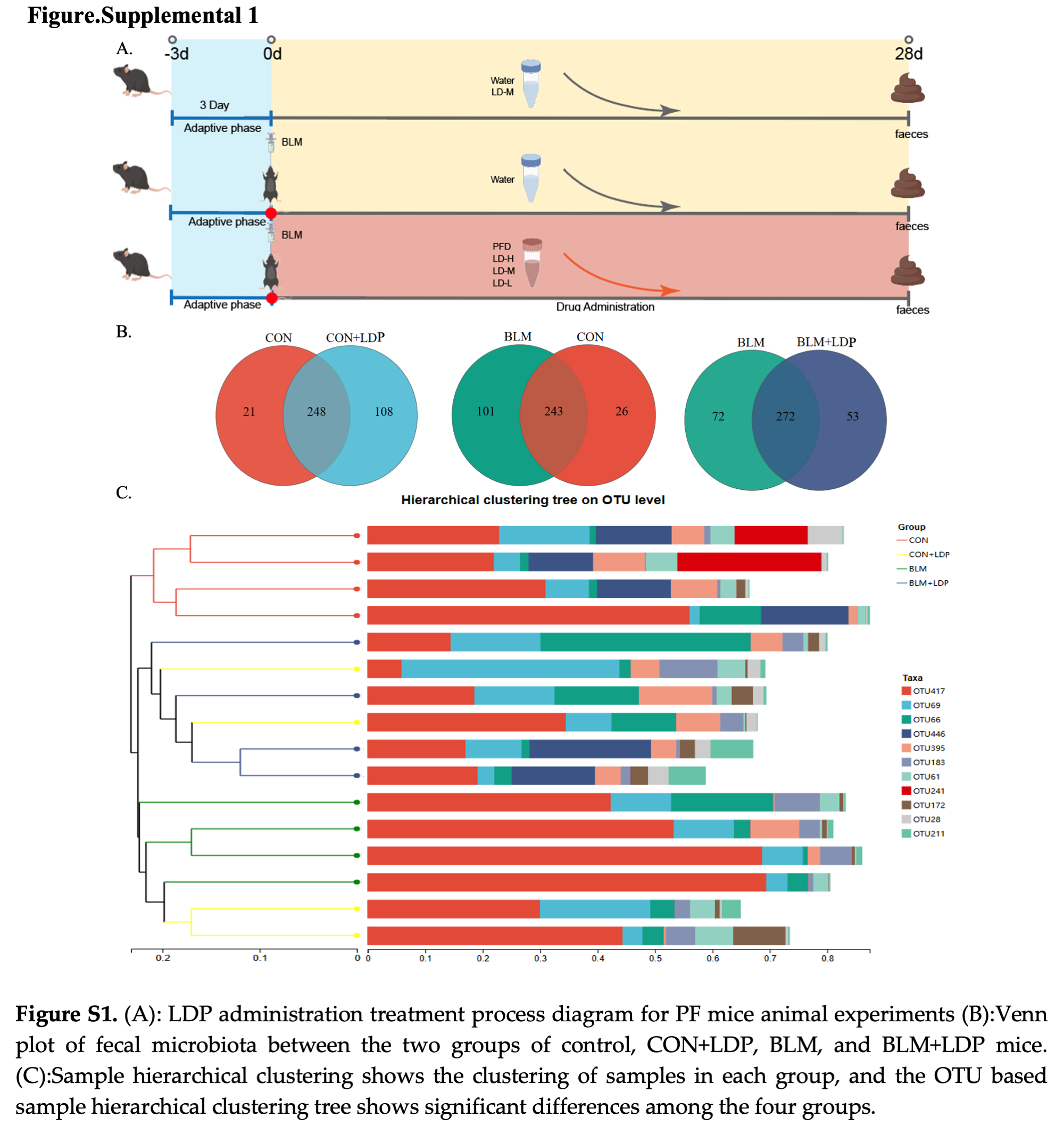

Supplement: Supplementary file 1 [file pharmaceuticals-19-00762-s001.zip › FIG.S1.tiff]

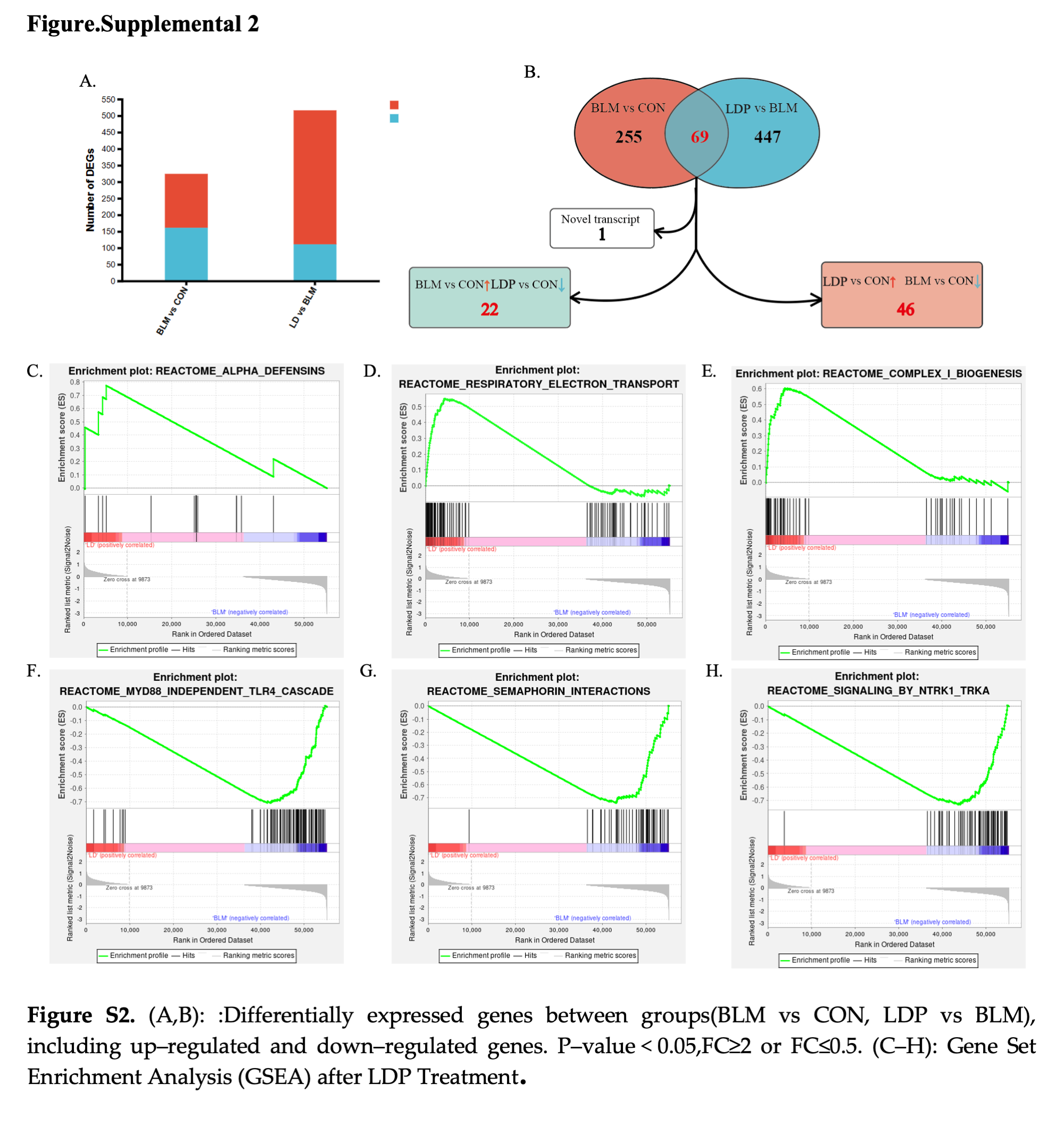

Supplement: Supplementary file 1 [file pharmaceuticals-19-00762-s001.zip › FIG.S2.tiff]

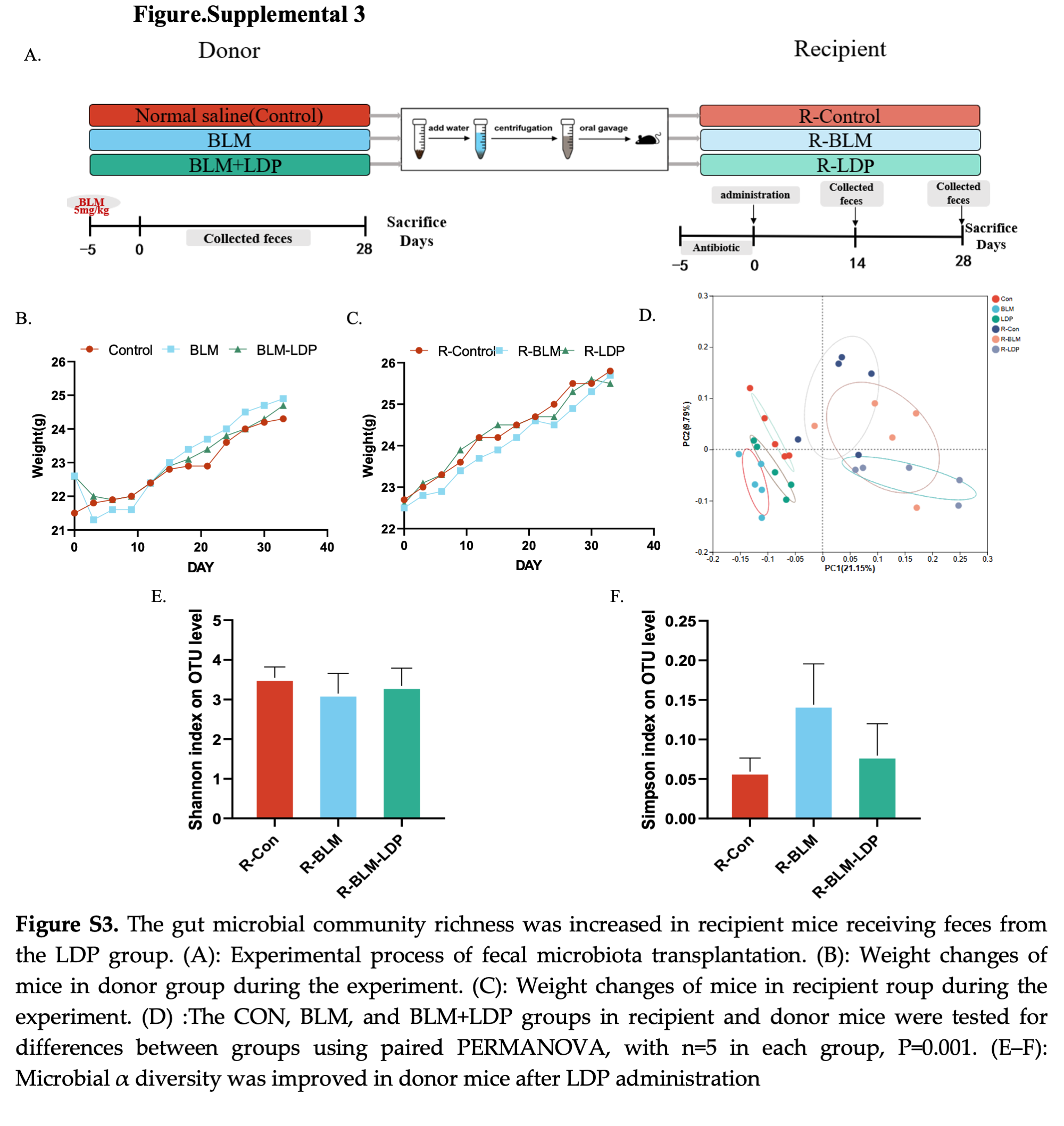

Supplement: Supplementary file 1 [file pharmaceuticals-19-00762-s001.zip › FIG.S3.tiff]

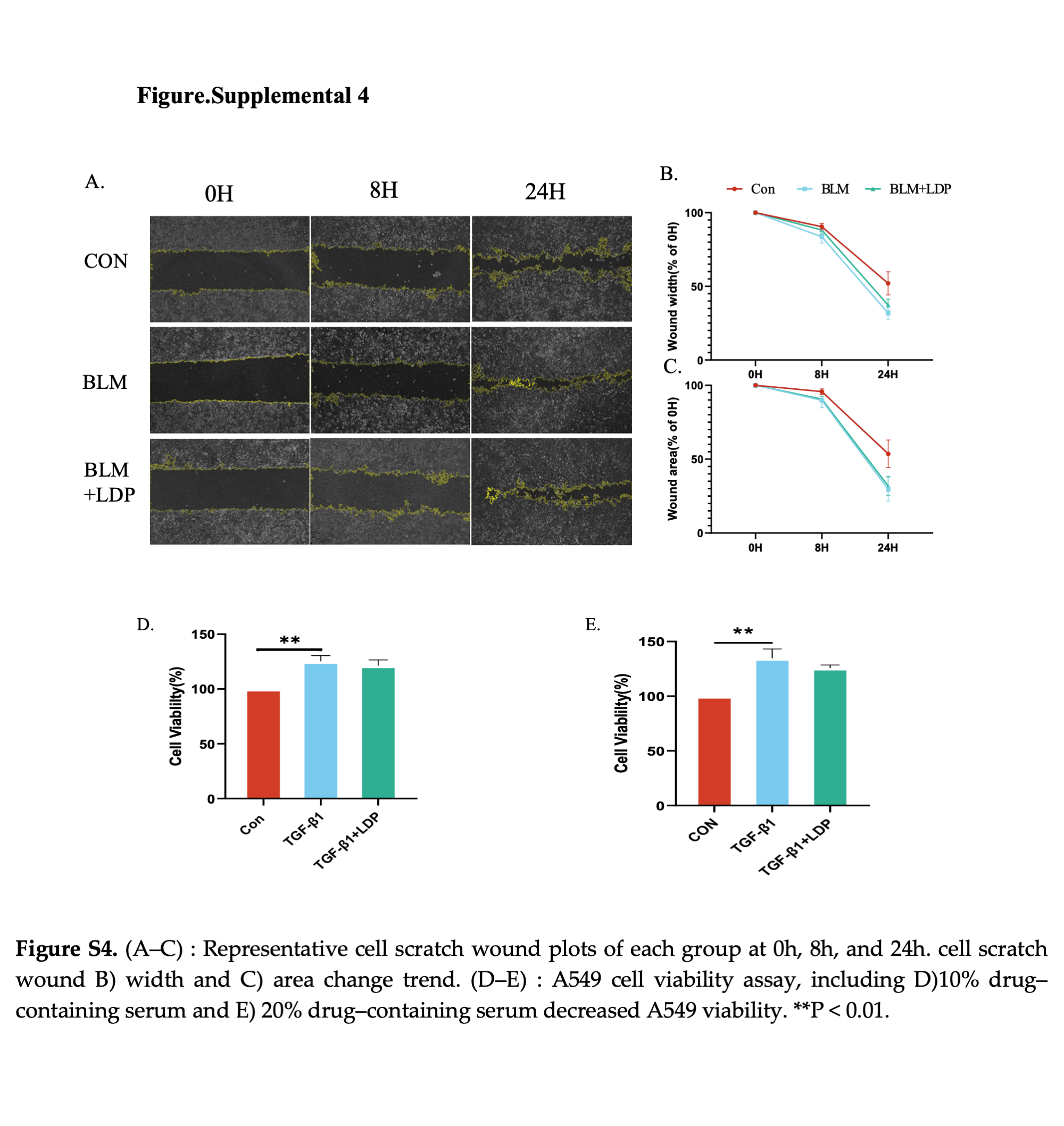

Supplement: Supplementary file 1 [file pharmaceuticals-19-00762-s001.zip › FIG.S4.tiff]
